# Supplementary material for: Fast-Track Discovery of SARS-CoV-2-Neutralizing Antibodies from Human B Cells by Direct Functional Screening
Source: Viruses. 2024 Feb 22;16(3):339. doi: 10.3390/v16030339 (PMC10975424; doi:10.3390/v16030339)
Supplement: Supplementary file 1 [file viruses-16-00339-s001.zip › Hillenbrand et al. Figure S1.pdf]

Supplemental data figure S1:

Sequences of SARS-CoV-2 variants used in this study are aligned using the multiple sequence alignment tool of Benchling. In order to increase surface expression, the SARS-CoV-2 spike protein was modified by a deletion of the furin cleavage site (aa682-685, “RRAR”, replaced by a single alanine) and by a C-terminal truncation of the last 17 amino acids, as described in Material & Methods. Mutations in respect to Wuhan strain are highlighted in red.

Page 1 Alpha (B.1.1.7), Beta (B.1.351), Gamma (B.1.1.248), Delta (B.1.617.2), Delta plus (B....

```
                              1                                                50
Wuhan                         MFVFLVLLPLVSSQCVNLTTRTQLPPAYTNSFTRGVYYPDKVFRSSVLHS
Alpha (B.1.1.7)               MFVFLVLLPLVSSQCVNLTTRTQLPPAYTNSFTRGVYYPDKVFRSSVLHS
Beta (B.1.351)                MFVFLVLLPLVSSQCVNFTTRTQLPPAYTNSFTRGVYYPDKVFRSSVLHS
Gamma (B.1.1.248)             MFVFLVLLPLVSSQCVNFTNRTQLPSAYTNSFTRGVYYPDKVFRSSVLHS
Delta (B.1.617.2)             MFVFLVLLPLVSSQCVNLRTRTQLPPAYTNSFTRGVYYPDKVFRSSVLHS
Delta plus (B.1.617.2 / AY.1) MFVFLVLLPLVSSQCVNLRTRTQLPPAYTNSFTRGVYYPDKVFRSSVLHS
Epsilon (B.1.429)             MFVFLVLLPLVSIQCVNLTTRTQLPPAYTNSFTRGVYYPDKVFRSSVLHS
Iota (B.1.526)                MFVFFVLLPLVSSQCVNLTTRTQLPPAYTNSFTRGVYYPDKVFRSSVLHS
Kappa (B.1.617.1)             MFVFLVLLPLVSSQCVNLTTRTQLPPAYTNSFTRGVYYPDKVFRSSVLHS
Lambda (C.37)                 MFVFLVLLPLVSSQCVNLTTRTQLPPAYTNSFTRGVYYPDKVFRSSVLHS
Omicron (B.1.1.529)           MFVFLVLLPLVSSQCVNLTTRTQLPPAYTNSFTRGVYYPDKVFRSSVLHS

..............................................................................

                              51                                              100
Wuhan                         TQDLFLPFFSNVTWFHAIHVSGTNGTKRFDNPVLPFNDGVYFASTEKSNI
Alpha (B.1.1.7)               TQDLFLPFFSNVTWFHAI--SGTNGTKRFDNPVLPFNDGVYFASTEKSNI
Beta (B.1.351)                TQDLFLPFFSNVTWFHAIHVSGTNGTKRFANPVLPFNDGVYFASTEKSNI
Gamma (B.1.1.248)             TQDLFLPFFSNVTWFHAIHVSGTNGTKRFDNPVLPFNDGVYFASTEKSNI
Delta (B.1.617.2)             TQDLFLPFFSNVTWFHAIHVSGTNGTKRFDNPVLPFNDGVYFASTEKSNI
Delta plus (B.1.617.2 / AY.1) TQDLFLPFFSNVTWFHAIHVSGTNGTKRFDNPVLPFNDGVYFASIEKSNI
Epsilon (B.1.429)             TQDLFLPFFSNVTWFHAIHVSGTNGTKRFDNPVLPFNDGVYFASTEKSNI
Iota (B.1.526)                TQDLFLPFFSNVTWFHAIHVSGTNGTKRFDNPVLPFNDGVYFASIEKSNI
Kappa (B.1.617.1)             TQDLFLPFFSNVTWFHAIHVSGTNGTKRFDNPVLPFNDGVYFASTEKSNI
Lambda (C.37)                 TQDLFLPFFSNVTWFHAIHVSGTNVIKRFDNPVLPFNDGVYFASTEKSNI
Omicron (B.1.1.529)           TQDLFLPFFSNVTWFHVI--SGTNGTKRFDNPVLPFNDGVYFASIEKSNI

..............................................................................

                              101                                             150
Wuhan                         IRGWIFGTTLDSKTQSLLIVNNATNVVIKVCEFQFCNDPFLGVYYHKNNK
Alpha (B.1.1.7)               IRGWIFGTTLDSKTQSLLIVNNATNVVIKVCEFQFCNDPFLGVY-HKNNK
Beta (B.1.351)                IRGWIFGTTLDSKTQSLLIVNNATNVVIKVCEFQFCNDPFLGVYYHKNNK
Gamma (B.1.1.248)             IRGWIFGTTLDSKTQSLLIVNNATNVVIKVCEFQFCNYPFLGVYYHKNNK
Delta (B.1.617.2)             IRGWIFGTTLDSKTQSLLIVNNATNVVIKVCEFQFCNDPFLDVYYHKNNK
Delta plus (B.1.617.2 / AY.1) IRGWIFGTTLDSKTQSLLIVNNATNVVIKVCEFQFCNDPFLGVYYHKNNK
Epsilon (B.1.429)             IRGWIFGTTLDSKTQSLLIVNNATNVVIKVCEFQFCNDPFLGVYYHKNNK
Iota (B.1.526)                IRGWIFGTTLDSKTQSLLIVNNATNVVIKVCEFQFCNDPFLGVYYHKNNK
Kappa (B.1.617.1)             IRGWIFGTTLDSKTQSLLIVNNATNVVIKVCEFQFCNDPFLDVYYHKNNK
Lambda (C.37)                 IRGWIFGTTLDSKTQSLLIVNNATNVVIKVCEFQFCNDPFLGVYYHKNNK
Omicron (B.1.1.529)           IRGWIFGTTLDSKTQSLLIVNNATNVVIKVCEFQFCNDPFLD---HKNNK

..............................................................................

                              151                                             200
Wuhan                         SWMESEFRVYSSANNCTFEYVSQPFLMDLEGKQGNFKNLREFVFKNIDGY
Alpha (B.1.1.7)               SWMESEFRVYSSANNCTFEYVSQPFLMDLEGKQGNFKNLREFVFKNIDGY
Beta (B.1.351)                SWMESEFRVYSSANNCTFEYVSQPFLMDLEGKQGNFKNLREFVFKNIDGY
Gamma (B.1.1.248)             SWMESEFRVYSSANNCTFEYVSQPFLMDLEGKQGNFKNLSEFVFKNIDGY
Delta (B.1.617.2)             SWMES--GVYSSANNCTFEYVSQPFLMDLEGKQGNFKNLREFVFKNIDGY
Delta plus (B.1.617.2 / AY.1) SWMES--GVYSSANNCTFEYVSQPFLMDLEGKQGNFKNLREFVFKNIDGY
Epsilon (B.1.429)             SCMESEFRVYSSANNCTFEYVSQPFLMDLEGKQGNFKNLREFVFKNIDGY
Iota (B.1.526)                SWMESEFRVYSSANNCTFEYVSQPFLMDLEGKQGNFKNLREFVFKNIDGY
Kappa (B.1.617.1)             SWMKSEFRVYSSANNCTFEYVSQPFLMDLEGKQGNFKNLREFVFKNIDGY
Lambda (C.37)                 SWMESEFRVYSSANNCTFEYVSQPFLMDLEGKQGNFKNLREFVFKNIDGY
Omicron (B.1.1.529)           SWMESEFRVYSSANNCTFEYVSQPFLMDLEGKQGNFKNLREFVFKNIDGY

..............................................................................
```

```
                               201                                              250
Wuhan                          FKIYSKHTPINL--VRDLPQGFSALEPLVDLPIGINITRFQTLLALHRSY
Alpha (B.1.1.7)                FKIYSKHTPINL--VRDLPQGFSALEPLVDLPIGINITRFQTLLALHRSY
Beta (B.1.351)                 FKIYSKHTPINL--VRGLPQGFSALEPLVDLPIGINITRFQT---LHRSY
Gamma (B.1.1.248)              FKIYSKHTPINL--VRDLPQGFSALEPLVDLPIGINITRFQTLLALHRSY
Delta (B.1.617.2)              FKIYSKHTPINL--VRDLPQGFSALEPLVDLPIGINITRFQTLLALHRSY
Delta plus (B.1.617.2 / AY.1)  FKIYSKHTPINL--VRDLPQGFSALEPLVDLPIGINITRFQTLLALHRSY
Epsilon (B.1.429)              FKIYSKHTPINL--VRDLPQGFSALEPLVDLPIGINITRFQTLLALHRSY
Iota (B.1.526)                 FKIYSKHTPINL--VRDLPQGFSALEPLVDLPIGINITRFQTLLALHRSY
Kappa (B.1.617.1)              FKIYSKHTPINL--VRDLPQGFSALEPLVDLPIGINITRFQTLLALHRSY
Lambda (C.37)                  FKIYSKHTPINL--VRDLPQGFSALEPLVDLPIGINITRFQTLLALHN--
Omicron (B.1.1.529)            FKIYSKHTPIIVREPEDLPQGFSALEPLVDLPIGINITRFQTLLALHRSY

..................................................................................

                               251                                              300
Wuhan                          LTPGDSSSGWTAGAAAYYVGYLQPRTFLLKYNENGTITDAVDCALDPLSE
Alpha (B.1.1.7)                LTPGDSSSGWTAGAAAYYVGYLQPRTFLLKYNENGTITDAVDCALDPLSE
Beta (B.1.351)                 LTPGDSSSGWTAGAAAYYVGYLQPRTFLLKYNENGTITDAVDCALDPLSE
Gamma (B.1.1.248)              LTPGDSSSGWTAGAAAYYVGYLQPRTFLLKYNENGTITDAVDCALDPLSE
Delta (B.1.617.2)              LTPGDSSSGWTAGAAAYYVGYLQPRTFLLKYNENGTITDAVDCALDPLSE
Delta plus (B.1.617.2 / AY.1)  LTPGDSSSGLTAGAAAYYVGYLQPRTFLLKYNENGTITDAVDCALDPLSE
Epsilon (B.1.429)              LTPGDSSSGWTAGAAAYYVGYLQPRTFLLKYNENGTITDAVDCALDPLSE
Iota (B.1.526)                 LTPGGSSSGWTAGAAAYYVGYLQPRTFLLKYNENGTITDAVDCALDPLSE
Kappa (B.1.617.1)              LTPGDSSSGWTAGAAAYYVGYLQPRTFLLKYNENGTITDAVDCALDPLSE
Lambda (C.37)                  -----SSSGWTAGAAAYYVGYLQPRTFLLKYNENGTITDAVDCALDPLSE
Omicron (B.1.1.529)            LTPGDSSSGWTAGAAAYYVGYLQPRTFLLKYNENGTITDAVDCALDPLSE

..................................................................................

                               301                                              350
Wuhan                          TKCTLKSFTVEKGIYQTSNFRVQPTESIVRFPNITNLCPFGEVFNATRFA
Alpha (B.1.1.7)                TKCTLKSFTVEKGIYQTSNFRVQPTESIVRFPNITNLCPFGEVFNATRFA
Beta (B.1.351)                 TKCTLKSFTVEKGIYQTSNFRVQPTESIVRFPNITNLCPFGEVFNATRFA
Gamma (B.1.1.248)              TKCTLKSFTVEKGIYQTSNFRVQPTESIVRFPNITNLCPFGEVFNATRFA
Delta (B.1.617.2)              TKCTLKSFTVEKGIYQTSNFRVQPTESIVRFPNITNLCPFGEVFNATRFA
Delta plus (B.1.617.2 / AY.1)  TKCTLKSFTVEKGIYQTSNFRVQPTESIVRFPNITNLCPFGEVFNATRFA
Epsilon (B.1.429)              TKCTLKSFTVEKGIYQTSNFRVQPTESIVRFPNITNLCPFGEVFNATRFA
Iota (B.1.526)                 TKCTLKSFTVEKGIYQTSNFRVQPTESIVRFPNITNLCPFGEVFNATRFA
Kappa (B.1.617.1)              TKCTLKSFTVEKGIYQTSNFRVQPTESIVRFPNITNLCPFGEVFNATRFA
Lambda (C.37)                  TKCTLKSFTVEKGIYQTSNFRVQPTESIVRFPNITNLCPFGEVFNATRFA
Omicron (B.1.1.529)            TKCTLKSFTVEKGIYQTSNFRVQPTESIVRFPNITNLCPFDEVFNATRFA

..................................................................................

                               351                                              400
Wuhan                          SVYAWNRKRISNCVADYSVLYNSASFSTFKCYGVSPTKLNDLCFTNVYAD
Alpha (B.1.1.7)                SVYAWNRKRISNCVADYSVLYNSASFSTFKCYGVSPTKLNDLCFTNVYAD
Beta (B.1.351)                 SVYAWNRKRISNCVADYSVLYNSASFSTFKCYGVSPTKLNDLCFTNVYAD
Gamma (B.1.1.248)              SVYAWNRKRISNCVADYSVLYNSASFSTFKCYGVSPTKLNDLCFTNVYAD
Delta (B.1.617.2)              SVYAWNRKRISNCVADYSVLYNSASFSTFKCYGVSPTKLNDLCFTNVYAD
Delta plus (B.1.617.2 / AY.1)  SVYAWNRKRISNCVADYSVLYNSASFSTFKCYGVSPTKLNDLCFTNVYAD
Epsilon (B.1.429)              SVYAWNRKRISNCVADYSVLYNSASFSTFKCYGVSPTKLNDLCFTNVYAD
Iota (B.1.526)                 SVYAWNRKRISNCVADYSVLYNSASFSTFKCYGVSPTKLNDLCFTNVYAD
Kappa (B.1.617.1)              SVYAWNRKRISNCVADYSVLYNSASFSTFKCYGVSPTKLNDLCFTNVYAD
Lambda (C.37)                  SVYAWNRKRISNCVADYSVLYNSASFSTFKCYGVSPTKLNDLCFTNVYAD
Omicron (B.1.1.529)            SVYAWNRKRISNCVADYSVLYNLAPFFTFKCYGVSPTKLNDLCFTNVYAD

..................................................................................
```

```
                                   401                                              450
Wuhan                              SFVIRGDEVRQIAPGQTGKIADYNYKLPDDFTGCVIAWNSNNLDSKVGGN
Alpha (B.1.1.7)                    SFVIRGDEVRQIAPGQTGKIADYNYKLPDDFTGCVIAWNSNNLDSKVGGN
Beta (B.1.351)                     SFVIRGDEVRQIAPGQTGNIADYNYKLPDDFTGCVIAWNSNNLDSKVGGN
Gamma (B.1.1.248)                  SFVIRGDEVRQIAPGQTGTIADYNYKLPDDFTGCVIAWNSNNLDSKVGGN
Delta (B.1.617.2)                  SFVIRGDEVRQIAPGQTGKIADYNYKLPDDFTGCVIAWNSNNLDSKVGGN
Delta plus (B.1.617.2 / AY.1)      SFVIRGDEVRQIAPGQTGNIADYNYKLPDDFTGCVIAWNSNNLDSKVGGN
Epsilon (B.1.429)                  SFVIRGDEVRQIAPGQTGKIADYNYKLPDDFTGCVIAWNSNNLDSKVGGN
Iota (B.1.526)                     SFVIRGDEVRQIAPGQTGKIADYNYKLPDDFTGCVIAWNSNNLDSKVGGN
Kappa (B.1.617.1)                  SFVIRGDEVRQIAPGQTGKIADYNYKLPDDFTGCVIAWNSNNLDSKVGGN
Lambda (C.37)                      SFVIRGDEVRQIAPGQTGKIADYNYKLPDDFTGCVIAWNSNNLDSKVGGN
Omicron (B.1.1.529)                SFVIRGDEVRQIAPGQTGNIADYNYKLPDDFTGCVIAWNSNKLDSKVSGN

..............................................................................................

                                   451                                              500
Wuhan                              YNYLYRLFRKSNLKPFERDISTEIYQAGSTPCNGVEGFNCYFPLQSYGFQ
Alpha (B.1.1.7)                    YNYLYRLFRKSNLKPFERDISTEIYQAGSTPCNGVEGFNCYFPLQSYGFQ
Beta (B.1.351)                     YNYLYRLFRKSNLKPFERDISTEIYQAGSTPCNGVKGFNCYFPLQSYGFQ
Gamma (B.1.1.248)                  YNYLYRLFRKSNLKPFERDISTEIYQAGSTPCNGVKGFNCYFPLQSYGFQ
Delta (B.1.617.2)                  YNYRYRLFRKSNLKPFERDISTEIYQAGSKPCNGVEGFNCYFPLQSYGFQ
Delta plus (B.1.617.2 / AY.1)      YNYRYRLFRKSNLKPFERDISTEIYQAGSKPCNGVEGFNCYFPLQSYGFQ
Epsilon (B.1.429)                  YNYRYRLFRKSNLKPFERDISTEIYQAGSTPCNGVEGFNCYFPLQSYGFQ
Iota (B.1.526)                     YNYLYRLFRKSNLKPFERDISTEIYQAGNTPCNGVKGFNCYFPLQSYGFQ
Kappa (B.1.617.1)                  YNYRYRLFRKSNLKPFERDISTEIYQAGSTPCNGVQGFNCYFPLQSYGFQ
Lambda (C.37)                      YNYQYRLFRKSNLKPFERDISTEIYQAGSTPCNGVEGFNCYSPLQSYGFQ
Omicron (B.1.1.529)                YNYLYRLFRKSNLKPFERDISTEIYQAGNKPCNGVAGFNCYFPLRSYSFR

..............................................................................................

                                   501                                              550
Wuhan                              PTNGVGYQPYRVVVLSFELLHAPATVCGPKKSTNLVKNKCVNFNFNGLTG
Alpha (B.1.1.7)                    PTYGVGYQPYRVVVLSFELLHAPATVCGPKKSTNLVKNKCVNFNFNGLTG
Beta (B.1.351)                     PTYGVGYQPYRVVVLSFELLHAPATVCGPKKSTNLVKNKCVNFNFNGLTG
Gamma (B.1.1.248)                  PTYGVGYQPYRVVVLSFELLHAPATVCGPKKSTNLVKNKCVNFNFNGLTG
Delta (B.1.617.2)                  PTNGVGYQPYRVVVLSFELLHAPATVCGPKKSTNLVKNKCVNFNFNGLTG
Delta plus (B.1.617.2 / AY.1)      PTNGVGYQPYRVVVLSFELLHAPATVCGPKKSTNLVKNKCVNFNFNGLTG
Epsilon (B.1.429)                  PTNGVGYQPYRVVVLSFELLHAPATVCGPKKSTNLVKNKCVNFNFNGLTG
Iota (B.1.526)                     PTNGVGYQPYRVVVLSFELLHAPATVCGPKKSTNLVKNKCVNFNFNGLTG
Kappa (B.1.617.1)                  PTNGVGYQPYRVVVLSFELLHAPATVCGPKKSTNLVKNKCVNFNFNGLTG
Lambda (C.37)                      PTNGVGYQPYRVVVLSFELLHAPATVCGPKKSTNLVKNKCVNFNFNGLTG
Omicron (B.1.1.529)                PTYGVGHQPYRVVVLSFELLHAPATVCGPKKSTNLVKNKCVNFNFNGLKG

..............................................................................................

                                   551                                              600
Wuhan                              TGVLTESNKKFLPFQQFGRDIADTTDAVRDPQTLEILDITPCSFGGVSVI
Alpha (B.1.1.7)                    TGVLTESNKKFLPFQQFGRDIDDTTDAVRDPQTLEILDITPCSFGGVSVI
Beta (B.1.351)                     TGVLTESNKKFLPFQQFGRDIADTTDAVRDPQTLEILDITPCSFGGVSVI
Gamma (B.1.1.248)                  TGVLTESNKKFLPFQQFGRDIADTTDAVRDPQTLEILDITPCSFGGVSVI
Delta (B.1.617.2)                  TGVLTESNKKFLPFQQFGRDIADTTDAVRDPQTLEILDITPCSFGGVSVI
Delta plus (B.1.617.2 / AY.1)      TGVLTESNKKFLPFQQFGRDIADTTDAVRDPQTLEILDITPCSFGGVSVI
Epsilon (B.1.429)                  TGVLTESNKKFLPFQQFGRDIADTTDAVRDPQTLEILDITPCSFGGVSVI
Iota (B.1.526)                     TGVLTESNKKFLPFQQFGRDIADTTDAVRDPQTLEILDITPCSFGGVSVI
Kappa (B.1.617.1)                  TGVLTESNKKFLPFQQFGRDIADTTDAVRDPQTLEILDITPCSFGGVSVI
Lambda (C.37)                      TGVLTESNKKFLPFQQFGRDIADTTDAVRDPQTLEILDITPCSFGGVSVI
Omicron (B.1.1.529)                TGVLTESNKKFLPFQQFGRDIADTTDAVRDPQTLEILDITPCSFGGVSVI

..............................................................................................
```

```
                              601                                              650
Wuhan                         TPGTNTSNQVAVLYQDVNCTEVPVAIHADQLTPTWRVYSTGSNVFQTRAG
Alpha (B.1.1.7)               TPGTNTSNQVAVLYQGVNCTEVPVAIHADQLTPTWRVYSTGSNVFQTRAG
Beta (B.1.351)                TPGTNTSNQVAVLYQGVNCTEVPVAIHADQLTPTWRVYSTGSNVFQTRAG
Gamma (B.1.1.248)             TPGTNTSNQVAVLYQGVNCTEVPVAIHADQLTPTWRVYSTGSNVFQTRAG
Delta (B.1.617.2)             TPGTNTSNQVAVLYQGVNCTEVPVAIHADQLTPTWRVYSTGSNVFQTRAG
Delta plus (B.1.617.2 / AY.1) TPGTNTSNQVAVLYQGVNCTEVPVAIHADQLTPTWRVYSTGSNVFQTRAG
Epsilon (B.1.429)             TPGTNTSNQVAVLYQDVNCTEVPVAIHADQLTPTWRVYSTGSNVFQTRAG
Iota (B.1.526)                TPGTNTSNQVAVLYQGVNCTEVPVAIHADQLTPTWRVYSTGSNVFQTRAG
Kappa (B.1.617.1)             TPGTNTSNQVAVLYQGVNCTEVPVAIHADQLTPTWRVYSTGSNVFQTRAG
Lambda (C.37)                 TPGTNTSNQVAVLYQGVNCTEVPVAIHADQLTPTWRVYSTGSNVFQTRAG
Omicron (B.1.1.529)           TPGTNTSNQVAVLYQGVNCTEVPVAIHADQLTPTWRVYSTGSNVFQTRAG

................................................................................

                              651                                              700
Wuhan                         CLIGAEHVNNSYECDIPIGAGICASYQTQTNSPA---SVASQSIIAYTMS
Alpha (B.1.1.7)               CLIGAEHVNNSYECDIPIGAGICASYQTQTNSHAGARSVASQSIIAYTMS
Beta (B.1.351)                CLIGAEHVNNSYECDIPIGAGICASYQTQTNSPA---SVASQSIIAYTMS
Gamma (B.1.1.248)             CLIGAEYVNNSYECDIPIGAGICASYQTQTNSPA---SVASQSIIAYTMS
Delta (B.1.617.2)             CLIGAEHVNNSYECDIPIGAGICASYQTQTNSRA---SVASQSIIAYTMS
Delta plus (B.1.617.2 / AY.1) CLIGAEHVNNSYECDIPIGAGICASYQTQTNSRA---SVASQSIIAYTMS
Epsilon (B.1.429)             CLIGAEHVNNSYECDIPIGAGICASYQTQTNSPA---SVASQSIIAYTMS
Iota (B.1.526)                CLIGAEHVNNSYECDIPIGAGICASYQTQTNSPA---SVASQSIIAYTMS
Kappa (B.1.617.1)             CLIGAEHVNNSYECDIPIGAGICASYQTQTNSRA---SVASQSIIAYTMS
Lambda (C.37)                 CLIGAEHVNNSYECDIPIGAGICASYQTQTNSPA---SVASQSIIAYTMS
Omicron (B.1.1.529)           CLIGAEYVNNSYECDIPIGAGICASYQTQTKSHA---SVASQSIIAYTMS

................................................................................

                              701                                              750
Wuhan                         LGAENSVAYSNNSIAIPTNFTISVTTEILPVSMTKTSVDCTMYICGDSTE
Alpha (B.1.1.7)               LGAENSVAYSNNSIAIPINFTISVTTEILPVSMTKTSVDCTMYICGDSTE
Beta (B.1.351)                LGVENSVAYSNNSIAIPTNFTISVTTEILPVSMTKTSVDCTMYICGDSTE
Gamma (B.1.1.248)             LGAENSVAYSNNSIAIPTNFTISVTTEILPVSMTKTSVDCTMYICGDSTE
Delta (B.1.617.2)             LGAENSVAYSNNSIAIPTNFTISVTTEILPVSMTKTSVDCTMYICGDSTE
Delta plus (B.1.617.2 / AY.1) LGAENSVAYSNNSIAIPTNFTISVTTEILPVSMTKTSVDCTMYICGDSTE
Epsilon (B.1.429)             LGAENSVAYSNNSIAIPTNFTISVTTEILPVSMTKTSVDCTMYICGDSTE
Iota (B.1.526)                LGVENSVAYSNNSIAIPTNFTISVTTEILPVSMTKTSVDCTMYICGDSTE
Kappa (B.1.617.1)             LGAENSVAYSNNSIAIPTNFTISVTTEILPVSMTKTSVDCTMYICGDSTE
Lambda (C.37)                 LGAENSVAYSNNSIAIPTNFTISVTTEILPVSMTKTSVDCTMYICGDSTE
Omicron (B.1.1.529)           LGAENSVAYSNNSIAIPTNFTISVTTEILPVSMTKTSVDCTMYICGDSTE

................................................................................

                              751                                              800
Wuhan                         CSNLLLQYGSFCTQLNRALTGIAVEQDKNTQEVFAQVKQIYKTPPIKDFG
Alpha (B.1.1.7)               CSNLLLQYGSFCTQLNRALTGIAVEQDKNTQEVFAQVKQIYKTPPIKDFG
Beta (B.1.351)                CSNLLLQYGSFCTQLNRALTGIAVEQDKNTQEVFAQVKQIYKTPPIKDFG
Gamma (B.1.1.248)             CSNLLLQYGSFCTQLNRALTGIAVEQDKNTQEVFAQVKQIYKTPPIKDFG
Delta (B.1.617.2)             CSNLLLQYGSFCTQLNRALTGIAVEQDKNTQEVFAQVKQIYKTPPIKDFG
Delta plus (B.1.617.2 / AY.1) CSNLLLQYGSFCTQLNRALTGIAVEQDKNTQEVFAQVKQIYKTPPIKDFG
Epsilon (B.1.429)             CSNLLLQYGSFCTQLNRALTGIAVEQDKNTQEVFAQVKQIYKTPPIKDFG
Iota (B.1.526)                CSNLLLQYGSFCTQLNRALTGIAVEQDKNTQEVFAQVKQIYKTPPIKDFG
Kappa (B.1.617.1)             CSNLLLQYGSFCTQLNRALTGIAVEQDKNTQEVFAQVKQIYKTPPIKDFG
Lambda (C.37)                 CSNLLLQYGSFCTQLNRALTGIAVEQDKNTQEVFAQVKQIYKTPPIKDFG
Omicron (B.1.1.529)           CSNLLLQYGSFCTQLKRALTGIAVEQDKNTQEVFAQVKQIYKTPPIKYFG

................................................................................
```

```
                                   801                                              850
Wuhan                              GFNFSQILPDPSKPSKRSFIEDLLFNKVTLADAGFIKQYGDCLGDIAARD
Alpha (B.1.1.7)                    GFNFSQILPDPSKPSKRSFIEDLLFNKVTLADAGFIKQYGDCLGDIAARD
Beta (B.1.351)                     GFNFSQILPDPSKPSKRSFIEDLLFNKVTLADAGFIKQYGDCLGDIAARD
Gamma (B.1.1.248)                  GFNFSQILPDPSKPSKRSFIEDLLFNKVTLADAGFIKQYGDCLGDIAARD
Delta (B.1.617.2)                  GFNFSQILPDPSKPSKRSFIEDLLFNKVTLADAGFIKQYGDCLGDIAARD
Delta plus (B.1.617.2 / AY.1)      GFNFSQILPDPSKPSKRSFIEDLLFNKVTLADAGFIKQYGDCLGDIAARD
Epsilon (B.1.429)                  GFNFSQILPDPSKPSKRSFIEDLLFNKVTLADAGFIKQYGDCLGDIAARD
Iota (B.1.526)                     GFNFSQILPDPSKPSKRSFIEDLLFNKVTLADAGFIKQYGDCLGDIAARD
Kappa (B.1.617.1)                  GFNFSQILPDPSKPSKRSFIEDLLFNKVTLADAGFIKQYGDCLGDIAARD
Lambda (C.37)                      GFNFSQILPDPSKPSKRSFIEDLLFNKVTLADAGFIKQYGDCLGDIAARD
Omicron (B.1.1.529)                GFNFSQILPDPSKPSKRSFIEDLLFNKVTLADAGFIKQYGDCLGDIAARD
```

....

```
                                   851                                              900
Wuhan                              LICAQKFNGLTVLPPLLTDEMIAQYTSALLAGTITSGWTFGAGAALQIPF
Alpha (B.1.1.7)                    LICAQKFNGLTVLPPLLTDEMIAQYTSALLAGTITSGWTFGAGAALQIPF
Beta (B.1.351)                     LICAQKFNGLTVLPPLLTDEMIAQYTSALLAGTITSGWTFGAGAALQIPF
Gamma (B.1.1.248)                  LICAQKFNGLTVLPPLLTDEMIAQYTSALLAGTITSGWTFGAGAALQIPF
Delta (B.1.617.2)                  LICAQKFNGLTVLPPLLTDEMIAQYTSALLAGTITSGWTFGAGAALQIPF
Delta plus (B.1.617.2 / AY.1)      LICAQKFNGLTVLPPLLTDEMIAQYTSALLAGTITSGWTFGAGAALQIPF
Epsilon (B.1.429)                  LICAQKFNGLTVLPPLLTDEMIAQYTSALLAGTITSGWTFGAGAALQIPF
Iota (B.1.526)                     LICAQKFNGLTVLPPLLTDEMIAQYTSALLAGTITSGWTFGAGAALQIPF
Kappa (B.1.617.1)                  LICAQKFNGLTVLPPLLTDEMIAQYTSALLAGTITSGWTFGAGAALQIPF
Lambda (C.37)                      LICAQKFNGLNVLPPLLTDEMIAQYTSALLAGTITSGWTFGAGAALQIPF
Omicron (B.1.1.529)                LICAQKFKGLTVLPPLLTDEMIAQYTSALLAGTITSGWTFGAGAALQIPF
```

....

```
                                   901                                              950
Wuhan                              AMQMAYRFNGIGVTQNVLYENQKLIANQFNSAIGKIQDSLSSTASALGKL
Alpha (B.1.1.7)                    AMQMAYRFNGIGVTQNVLYENQKLIANQFNSAIGKIQDSLSSTASALGKL
Beta (B.1.351)                     AMQMAYRFNGIGVTQNVLYENQKLIANQFNSAIGKIQDSLSSTASALGKL
Gamma (B.1.1.248)                  AMQMAYRFNGIGVTQNVLYENQKLIANQFNSAIGKIQDSLSSTASALGKL
Delta (B.1.617.2)                  AMQMAYRFNGIGVTQNVLYENQKLIANQFNSAIGKIQDSLSSTASALGKL
Delta plus (B.1.617.2 / AY.1)      AMQMAYRFNGIGVTQNVLYENQKLIANQFNSAIGKIQDSLSSTASALGKL
Epsilon (B.1.429)                  AMQMAYRFNGIGVTQNVLYENQKLIANQFNSAIGKIQDSLSSTASALGKL
Iota (B.1.526)                     AMQMAYRFNGIGVTQNVLYENQKLIANQFNSAIGKIQDSLSSTASALGKL
Kappa (B.1.617.1)                  AMQMAYRFNGIGVTQNVLYENQKLIANQFNSAIGKIQDSLSSTASALGKL
Lambda (C.37)                      AMQMAYRFNGIGVTQNVLYENQKLIANQFNSAIGKIQDSLSSTASALGKL
Omicron (B.1.1.529)                AMQMAYRFNGIGVTQNVLYENQKLIANQFNSAIGKIQDSLSSTASALGKL
```

....

```
                                   951                                             1000
Wuhan                              QDVVNQNAQALNTLVKQLSSNFGAISSVLNDILSRLDKVEAEVQIDRLIT
Alpha (B.1.1.7)                    QDVVNQNAQALNTLVKQLSSNFGAISSVLNDILARLDKVEAEVQIDRLIT
Beta (B.1.351)                     QDVVNQNAQALNTLVKQLSSNFGAISSVLNDILSRLDKVEAEVQIDRLIT
Gamma (B.1.1.248)                  QDVVNQNAQALNTLVKQLSSNFGAISSVLNDILSRLDKVEAEVQIDRLIT
Delta (B.1.617.2)                  QNVVNQNAQALNTLVKQLSSNFGAISSVLNDILSRLDKVEAEVQIDRLIT
Delta plus (B.1.617.2 / AY.1)      QNVVNQNAQALNTLVKQLSSNFGAISSVLNDILSRLDKVEAEVQIDRLIT
Epsilon (B.1.429)                  QDVVNQNAQALNTLVKQLSSNFGAISSVLNDILSRLDKVEAEVQIDRLIT
Iota (B.1.526)                     QDVVNQNAQALNTLVKQLSSNFGAISSVLNDILSRLDKVEAEVQIDRLIT
Kappa (B.1.617.1)                  QDVVNQNAQALNTLVKQLSSNFGAISSVLNDILSRLDKVEAEVQIDRLIT
Lambda (C.37)                      QDVVNQNAQALNTLVKQLSSNFGAISSVLNDILSRLDKVEAEVQIDRLIT
Omicron (B.1.1.529)                QDVVNHNAQALNTLVKQLSSKFGAISSVLNDIFSRLDKVEAEVQIDRLIT
```

....

```
                                 1001                                             1050
Wuhan                            GRLQSLQTYVTQQLIRAAEIRASANLAATKMSECVLGQSKRVDFCGKGYH
Alpha (B.1.1.7)                  GRLQSLQTYVTQQLIRAAEIRASANLAATKMSECVLGQSKRVDFCGKGYH
Beta (B.1.351)                   GRLQSLQTYVTQQLIRAAEIRASANLAATKMSECVLGQSKRVDFCGKGYH
Gamma (B.1.1.248)                GRLQSLQTYVTQQLIRAAEIRASANLAAIKMSECVLGQSKRVDFCGKGYH
Delta (B.1.617.2)                GRLQSLQTYVTQQLIRAAEIRASANLAATKMSECVLGQSKRVDFCGKGYH
Delta plus (B.1.617.2 / AY.1)    GRLQSLQTYVTQQLIRAAEIRASANLAATKMSECVLGQSKRVDFCGKGYH
Epsilon (B.1.429)                GRLQSLQTYVTQQLIRAAEIRASANLAATKMSECVLGQSKRVDFCGKGYH
Iota (B.1.526)                   GRLQSLQTYVTQQLIRAAEIRASANLAATKMSECVLGQSKRVDFCGKGYH
Kappa (B.1.617.1)                GRLQSLQTYVTQQLIRAAEIRASANLAATKMSECVLGQSKRVDFCGKGYH
Lambda (C.37)                    GRLQSLQTYVTQQLIRAAEIRASANLAATKMSECVLGQSKRVDFCGKGYH
Omicron (B.1.1.529)              GRLQSLQTYVTQQLIRAAEIRASANLAATKMSECVLGQSKRVDFCGKGYH

...............................................................................................

                                 1051                                             1100
Wuhan                            LMSFPQSAPHGVVFLHVTYVPAQEKNFTTAPAICHDGKAHFPREGVFVSN
Alpha (B.1.1.7)                  LMSFPQSAPHGVVFLHVTYVPAQEKNFTTAPAICHDGKAHFPREGVFVSN
Beta (B.1.351)                   LMSFPQSAPHGVVFLHVTYVPAQEKNFTTAPAICHDGKAHFPREGVFVSN
Gamma (B.1.1.248)                LMSFPQSAPHGVVFLHVTYVPAQEKNFTTAPAICHDGKAHFPREGVFVSN
Delta (B.1.617.2)                LMSFPQSAPHGVVFLHVTYVPAQEKNFTTAPAICHDGKAHFPREGVFVSN
Delta plus (B.1.617.2 / AY.1)    LMSFPQSAPHGVVFLHVTYVPAQEKNFTTAPAICHDGKAHFPREGVFVSN
Epsilon (B.1.429)                LMSFPQSAPHGVVFLHVTYVPAQEKNFTTAPAICHDGKAHFPREGVFVSN
Iota (B.1.526)                   LMSFPQSAPHGVVFLHVTYVPAQEKNFTTAPAICHDGKAHFPREGVFVSN
Kappa (B.1.617.1)                LMSFPQSAPHGVVFLHVTYVPAHEKNFTTAPAICHDGKAHFPREGVFVSN
Lambda (C.37)                    LMSFPQSAPHGVVFLHVTYVPAQEKNFTTAPAICHDGKAHFPREGVFVSN
Omicron (B.1.1.529)              LMSFPQSAPHGVVFLHVTYVPAQEKNFTTAPAICHDGKAHFPREGVFVSN

...............................................................................................

                                 1101                                             1150
Wuhan                            GTHWFVTQRNFYEPQIITTDNTFVSGNCDVVIGIVNNTVYDPLQPELDSF
Alpha (B.1.1.7)                  GTHWFVTQRNFYEPQIITTHNTFVSGNCDVVIGIVNNTVYDPLQPELDSF
Beta (B.1.351)                   GTHWFVTQRNFYEPQIITTDNTFVSGNCDVVIGIVNNTVYDPLQPELDSF
Gamma (B.1.1.248)                GTHWFVTQRNFYEPQIITTDNTFVSGNCDVVIGIVNNTVYDPLQPELDSF
Delta (B.1.617.2)                GTHWFVTQRNFYEPQIITTDNTFVSGNCDVVIGIVNNTVYDPLQPELDSF
Delta plus (B.1.617.2 / AY.1)    GTHWFVTQRNFYEPQIITTDNTFVSGNCDVVIGIVNNTVYDPLQPELDSF
Epsilon (B.1.429)                GTHWFVTQRNFYEPQIITTDNTFVSGNCDVVIGIVNNTVYDPLQPELDSF
Iota (B.1.526)                   GTHWFVTQRNFYEPQIITTDNTFVSGNCDVVIGIVNNTVYDPLQPELDSF
Kappa (B.1.617.1)                GTDWFVTQRNFYEPQIITTDNTFVSGNCDVVIGIVNNTVYDPLQPELDSF
Lambda (C.37)                    GTHWFVTQRNFYEPQIITTDNTFVSGNCDVVIGIVNNTVYDPLQPELDSF
Omicron (B.1.1.529)              GTHWFVTQRNFYEPQIITTDNTFVSGNCDVVIGIVNNTVYDPLQPELDSF

...............................................................................................

                                 1151                                             1200
Wuhan                            KEELDKYFKNHTSPDVDLGDISGINASVVNIQKEIDRLNEVAKNLNESLI
Alpha (B.1.1.7)                  KEELDKYFKNHTSPDVDLGDISGINASVVNIQKEIDRLNEVAKNLNESLI
Beta (B.1.351)                   KEELDKYFKNHTSPDVDLGDISGINASVVNIQKEIDRLNEVAKNLNESLI
Gamma (B.1.1.248)                KEELDKYFKNHTSPDVDLGDISGINASFVNIQKEIDRLNEVAKNLNESLI
Delta (B.1.617.2)                KEELDKYFKNHTSPDVDLGDISGINASVVNIQKEIDRLNEVAKNLNESLI
Delta plus (B.1.617.2 / AY.1)    KEELDKYFKNHTSPDVDLGDISGINASVVNIQKEIDRLNEVAKNLNESLI
Epsilon (B.1.429)                KEELDKYFKNHTSPDVDLGDISGINASVVNIQKEIDRLNEVAKNLNESLI
Iota (B.1.526)                   KEELDKYFKNHTSPDVDLGDISGINASVVNIQKEIDRLNEVAKNLNESLI
Kappa (B.1.617.1)                KEELDKYFKNHTSPDVDLGDISGINASVVNIQKEIDRLNEVAKNLNESLI
Lambda (C.37)                    KEELDKYFKNHTSPDVDLGDISGINASVVNIQKEIDRLNEVAKNLNESLI
Omicron (B.1.1.529)              KEELDKYFKNHTSPDVDLGDISGINASVVNIQKEIDRLNEVAKNLNESLI

...............................................................................................
```

```
                               1201                                              1250
Wuhan                          DLQELGKYEQYIKWPWYIWLGFIAGLIAIVMVTIMLCCMTSCCSCLKGCC
Alpha (B.1.1.7)                DLQELGKYEQYIKWPWYIWLGFIAGLIAIVMVTIMLCCMTSCCSCLKGCC
Beta (B.1.351)                 DLQELGKYEQYIKWPWYIWLGFIAGLIAIVMVTIMLCCMTSCCSCLKGCC
Gamma (B.1.1.248)              DLQELGKYEQYIKWPWYIWLGFIAGLIAIVMVTIMLCCMTSCCSCLKGCC
Delta (B.1.617.2)              DLQELGKYEQYIKWPWYIWLGFIAGLIAIVMVTIMLCCMTSCCSCLKGCC
Delta plus (B.1.617.2 / AY.1) DLQELGKYEQYIKWPWYIWLGFIAGLIAIVMVTIMLCCMTSCCSCLKGCC
Epsilon (B.1.429)              DLQELGKYEQYIKWPWYIWLGFIAGLIAIVMVTIMLCCMTSCCSCLKGCC
Iota (B.1.526)                 DLQELGKYEQYIKWPWYIWLGFIAGLIAIVMVTIMLCCMTSCCSCLKGCC
Kappa (B.1.617.1)              DLQELGKYEQYIKWPWYIWLGFIAGLIAIVMVTIMLCCMTSCCSCLKGCC
Lambda (C.37)                  DLQELGKYEQYIKWPWYIWLGFIAGLIAIVMVTIMLCCMTSCCSCLKGCC
Omicron (B.1.1.529)            DLQELGKYEQYIKWPWYIWLGFIAGLIAIVMVTIMLCCMTSCCSCLKGCC
```

....

```
                               12511258
Wuhan                          SCGSCCKF
Alpha (B.1.1.7)                SCGSCCKF
Beta (B.1.351)                 SCGSCCKF
Gamma (B.1.1.248)              SCGSCCKF
Delta (B.1.617.2)              SCGSCCKF
Delta plus (B.1.617.2 / AY.1) SCGSCCKF
Epsilon (B.1.429)              SCGSCCKF
Iota (B.1.526)                 SCGSCCKF
Kappa (B.1.617.1)              SCGSCCKF
Lambda (C.37)                  SCGSCCKF
Omicron (B.1.1.529)            SCGSCCKF
```

....
