# Supplementary material for: Fast-Track Discovery of SARS-CoV-2-Neutralizing Antibodies from Human B Cells by Direct Functional Screening
Source: Viruses. 2024 Feb 22;16(3):339. doi: 10.3390/v16030339 (PMC10975424; doi:10.3390/v16030339)

Supplemental data figure S2:

Sorted clones after the second selection round were once more confirmed by analytical flow cytometry for RBD-binding and absence of huACE2 binding, a read-out, which we used as a surrogate for neutralization. The plot shows examples for clones displaying a non-neutralizing antibody (binding both RBD and huACE2), a neutralizing antibody (CoVAb 36/MTX-COVAB binding only RBD is shown) and the negative control antibody (24C03), which binds neither RBD nor huACE2.

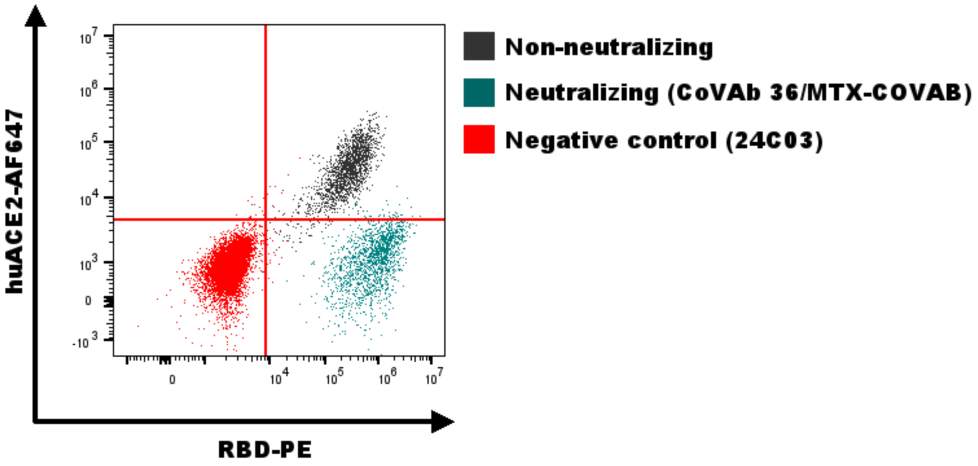

Supplement: Supplementary file 1 [file viruses-16-00339-s001.zip › Hillenbrand et al. Figure S2.pdf]
