# Supplementary material for: Fast-Track Discovery of SARS-CoV-2-Neutralizing Antibodies from Human B Cells by Direct Functional Screening
Source: Viruses. 2024 Feb 22;16(3):339. doi: 10.3390/v16030339 (PMC10975424; doi:10.3390/v16030339)
Supplement: Supplementary file 1 [file viruses-16-00339-s001.zip › Hillenbrand et al. Table S1.pdf]

Supplemental data Table S1:

Table S1: CDR Sequences of the four lead candidates

	**Light chain**			**Heavy chain**		
**mAbs**	CDR1	CDR2	CDR3	CDR1	CDR2	CDR3
**CoVAb 5**	RASQSISSWLA	KASSLES	QQYNIYSWT	DYYMS	YITSSSSYTNYADSVKG	APGIESSSWKQGVGY
**CoVAb 13**	QASQGISNYLN	DASNLET	QQYDNLPRYT	SNYMN	VIYSGGSTFQADSVKG	DRGDAFDI
**CoVAb 36**	TGTSSDVGGYNYVS	EVTSRPS	SSYTTSNTLGGV	SNYMS	VIYSGGITYFADSVKG	DRRLPHYYYGLDV
**CoVAb 47**	RASQGISNYLA	ASSTLQS	QHLNSYPH	SNYMS	IIYPGGSRFYADSVKG	DLYYYGMDV
